# Long-term results of minimally invasive transanal surgery for rectal tumors in 249 consecutive patients

**DOI:** 10.1007/s00595-022-02570-z

**Published:** 2022-08-12

**Authors:** Kotaro Maeda, Yoshikazu Koide, Hidetoshi Katsuno, Yosuke Tajima, Tsunekazu Hanai, Koji Masumori, Hiroshi Matsuoka, Miho Shiota

**Affiliations:** 1 Department of Surgery, Medical Corporation Kenikukai Shonan Keiiku Hospital, 4360 Endo, Fujisawa, Kanagawa 252-0816 Japan; 2 Department of Surgery, Fujita Health University Hospital, Toyoake, 470-1192 Japan; 3 Department of Surgery, Fujita Health University Okazaki Medical Center, Okazaki, 444-0827 Japan; 4 Department of Surgery, Kaisei Hospital, Sakaide, 657-0068 Japan

**Keywords:** Local excision, Transanal surgery, Minimally invasive transanal surgery, Rectal cancer, Rectal tumor

## Abstract

**Purpose:**

To delineate the long-term results of minimally invasive transanal surgery (MITAS) for selected rectal tumors.

**Methods:**

We analyzed data, retrospectively, on consecutive patients who underwent MITAS between 1995 and 2015, to establish the feasibility, excision quality, and perioperative and oncological outcomes of this procedure.

**Results:**

MITAS was performed on 243 patients. The final histology included 142 cancers, 47 adenomas, and 52 neuroendocrine tumors (NET G1). A positive margin of 1.6% and 100% *en bloc* resection were achieved. The mean operative time was 27.4 min. Postoperative morbidity occurred in 7% of patients, with 0% mortality. The median follow-up was 100 months (up to ≥ 5 years or until death in 91.8% of patients). Recurrence developed in 2.9% of the patients. The 10-year overall survival rate was 100% for patients with NET G1 and 80.3% for those with cancer. The 5-year DFS was 100% for patients with Tis cancer, 90.6% for those with T1 cancer, and 87.5% for those with T2 or deeper cancers. MITAS for rectal tumors ≥ 3 cm resulted in perioperative and oncologic outcomes equivalent to those for tumors < 3 cm.

**Conclusion:**

MITAS is feasible for the local excision (LE) of selected rectal tumors, including tumors ≥ 3 cm. It reduces operative time and secures excision quality and long-term oncological outcomes.

## Introduction

Local excision (LE) is being performed increasingly for early rectal cancer [1, 2]. While oncological outcomes following LE of T1 rectal tumors are improving [2], the associated local recurrence rates are still consistently higher than those after radical resection [1, 3–5]. LE is recommended for carefully selected patients with rectal cancer cT1N0 without high-risk characteristics [3]. However, distinguishing the depth of invasion (Tis, T1, or T2) may be difficult with magnetic resonance imaging (MRI), although endoscopic ultrasound can be used as a complementary staging tool in certain situations [3]. The clinical criteria for local treatment typically include small (< 3 cm) adenocarcinomas limited to < 30% of the rectal circumference [3, 6]. It is difficult to diagnose high-risk characteristics histologically by preoperative biopsy and even if the preoperative biopsy reveals a benign rectal polyp, subsequent upstaging to rectal cancer is common [7–9]. Therefore, LE for rectal tumors should be performed based on the following criteria: preoperative biopsy finding of adenoma, identification of well-differentiated or moderately differentiated adenocarcinomas, and accurate identification of the depth of invasion [7] in addition to proposed tumor size [3, 6]. If pathological examination after LE reveals significant risk factors, subsequent radical resection is typically recommended [3, 6, 9–11], but its significance is unclear [12]. Furthermore, on subsequent total mesorectal excision (TME) after LE, an increased risk of abdominoperineal resection (APR) and worsening quality of TME have been reported [13, 14].

LE can be performed as conventional LE or using transanal endoscopic platforms such as transanal endoscopic microsurgery (TEMS), transanal minimally invasive surgery (TAMIS), or endoscopic submucosal dissection (ESD) [3, 6, 8–10]. The characteristics and superiority of each procedure have been analyzed with respect to tumor location, *en bloc* resection rate, surgical margin negative rate (R0), procedure time, complication rate, local recurrence, and survival [3, 6, 8–10, 15, 16]. However, long-term outcomes after LE are rarely studied, especially for tumors ≥ 3 cm in diameter [17, 18].

We analyzed the tumor characteristics and perioperative and long-term outcomes following our minimally invasive transanal surgery (MITAS) LE approach. We developed this approach using a specially designed anal retractor, a stapler device, and several modified surgical techniques under direct vision for selected rectal tumors, especially those ≥ 3 cm in diameter, with the results of subsequent rectal resection, to clarify the importance of MITAS LE for rectal tumors.

## Methods

The study population comprised consecutive patients undergoing MITAS LE between October, 1995 and December, 2015, at the Fujita Health University Hospital. All the patients were prospectively registered and retrospectively reviewed by their medical records.

LE was selected based on previously described findings in adenoma and cancer [7]. Neuroendocrine tumors (NET G1) ≤ 1.5 cm in diameter, localized tumors with undetermined histology, and tumors with margin positive or recurrent adenoma after endoscopic resection were also indicated for MITAS LE. Tumors including adenoma, cancer, and all the other tumors were unsuitable for *en bloc* endoscopic resection. MITAS LE has been performed selectively since October 2001, for patients with cT2 or cT3 cancers, who were unwilling or unfit to undergo radical surgery, and for symptomatic patients with unresectable liver metastases. MITAS LE was performed for tumors ≥ 3 cm in diameter, but tumors surrounding the bowel wall were excluded. Conventional LE was performed principally for tumors located < 5 cm from the anal verge (AV); otherwise, MITAS LE was selected.

The tumor location was identified by rigid proctoscopy or digital examination, and the distance from the AV to the distal part of the tumor or scar was measured. Macroscopic type was assessed during colonoscopy and classified according to the Japanese Classification of Colorectal, Appendiceal, and Anal Carcinoma and Association of Coloproctology of Great Britain and Ireland’s (ACPGBI) position statement [19, 20]. When two tumors were removed simultaneously by MITAS LE, the larger tumor or the tumor with a greater depth of invasion was listed for the patient.

**Fig. 1:**
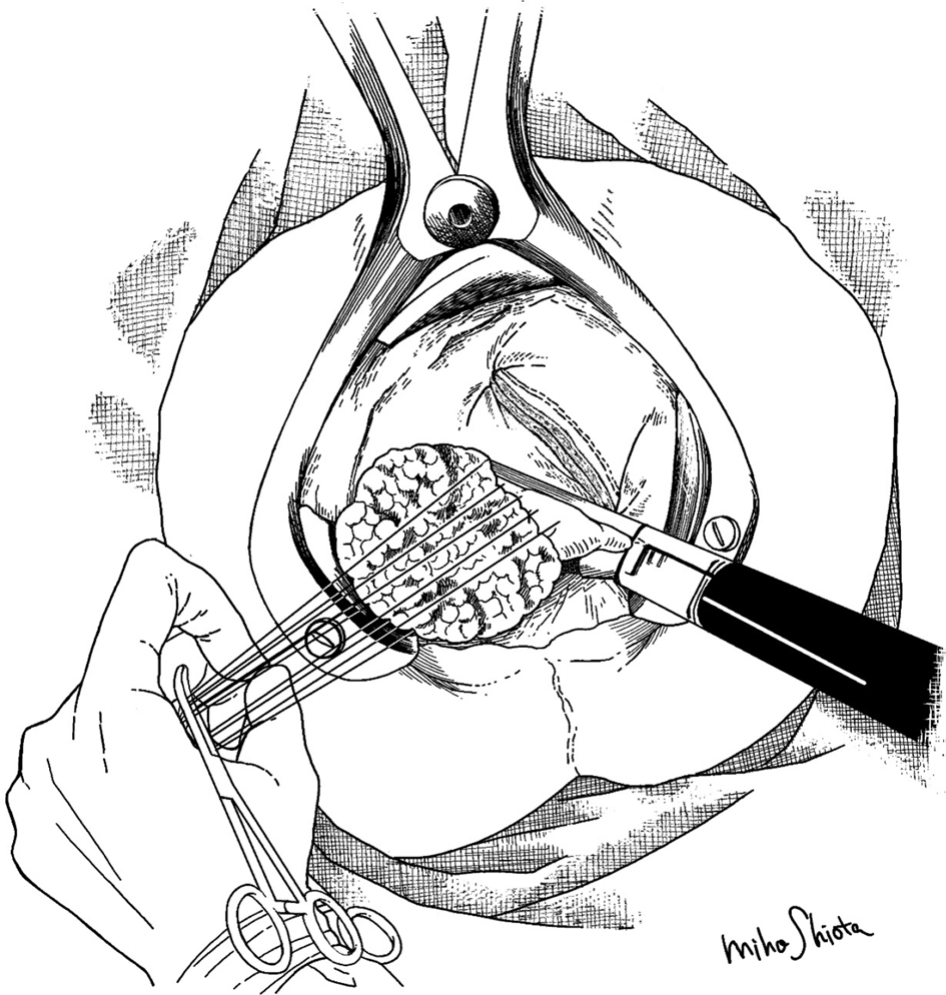
Minimally invasive transanal surgery procedure. The invaginated rectum with the tumor is excised and anastomosed simultaneously by a stapler while fully retracting the rectum with retraction stitches, distally or laterally, after shortening the rectum using a new retractor

Bowel preparation, anesthesia, and positioning were carried out as described previously [7, 21–23]. The procedure involved inserting an originally designed E- or F-type anal retractor (a modified K-type anal retractor [24], Yufu Itonaga Co. Ltd.) (Fig. 1) into the rectum, connected to an Octopus retractor holder (long type, 22 in, Mednosbro AG), using the shortening or roll-in technique, intussusception, and sufficient retraction stitches to retract the tumor fully and pull the rectum down [7].

Prior to performing LE with the stapler, the rectum on and around the stapler line was swabbed routinely. ENDO TA or ENDO GIA (Medtronic Japan Co Ltd) was used for excision and anastomosis when fully retracting the rectum with retraction stitches distally or laterally (Fig. 1). All operations were performed by the same surgeon (KM).

Immediately after MITAS LE, the staplers were removed carefully from the specimen, mounted on a cork specimen board, and measured before formalin fixation. The pathology analysis included histological typing, tumor differentiation, grade of carcinoma invasion, presence or absence of lymphatic or vascular vessel invasion, and a circumferential and deep (surgical) margin with depth of excision. Carcinoma invasion in a pT1 tumor was graded according to the criteria defined by Kikuchi et al. [25]; T1 tumors with depth of invasion sm1 were considered pT1a, and those with sm2 and 3 were considered pT1b [19, 25] or graded by depth of invasion < 1000 (pT1a) or ≥ 1000 (pT1b) micrometers according to the Japanese Classification of Colorectal, Appendiceal, and Anal Carcinoma [19]. The surgical margin was defined as positive when the tumor was present at the circumferential and deep margin, according to the pathology report. A T1 tumor was classified as high risk when a pT1b tumor, poor tumor differentiation, or vessel invasion was present [19, 26, 27]; otherwise, it was classified as low risk. Patients with high-risk T1 or T2 tumors or deeper tumors were advised to undergo additional surgery for possible lymph node metastasis [19, 26, 27]. Histology was identified from endoscopically resected specimens of tumors with a positive margin after endoscopic resection.

Patients were followed up at the outpatient clinic, at 3, 6, and 12 months and every 1–2 years thereafter, as described previously [7]; for a minimum of 5 years, or until death, whichever occurred first. Follow-up data were obtained from the medical records of our clinic or referral hospital or by telephone interviews. Local recurrence was defined as a lesion on and around the suture line.

The primary endpoints were feasibility, excision quality, and oncologic outcomes with survival by MITAS LE, especially for tumors ≥ 3 cm in diameter. Resection quality was determined by *en bloc* resection rates and margin positivity. Secondary endpoints included operative outcomes and perioperative morbidity and mortality. APR rates and quality of the excised specimens were evaluated for patients undergoing subsequent surgery after LE. Furthermore, risk factors for recurrence were studied in patients with high-risk T1 tumors and T2 or deeper tumors without liver metastasis.

Statistical significance was established using the Mann–Whitney U test for continuous variables and the chi-square test or Fisher’s exact test for categorical variables. Differences were considered significant for *P* < 0.05. Kaplan–Meier survival analyses were used to evaluate the overall survival (OS), disease-free survival (DFS), and local recurrence-free survival rates. The log-rank test was used for comparisons. All statistical analyses were performed using IBM SDPSS statistics v.27 (IBM Japan Inc., Tokyo, Japan).

## Results

During the 230-month study period, 249 patients underwent MITAS LE. None of the patients received preoperative chemotherapy or chemoradiotherapy for rectal tumors. MITAS LE could not be completed in 6 of the 249 patients (2.4%) and Table 1 presents the characteristics of the remaining 243 patients who underwent MITAS LE successfully. The final histology was cancer in 142 patients (well- and moderately differentiated adenocarcinoma in 127 and 15, respectively), adenoma in 42 patients, NET G1 in 52 patients, schwannoma in 1patient, and mucosa-associated lymphoid tissue lymphoma in 1 patient. Of the 46 pT1 tumors, 13 were diagnosed as low-risk T1 and the remaining 33, as high risk. Of the 22 patients undergoing MITAS LE as additional surgery, adenoma was confirmed in one patient with recurrence; otherwise, no residual tumor or vessel invasion was observed. The median distance from the AV to the tumor or scar was 8 cm and nine tumors were located > 16 cm from the AV (distal sigmoid colon). One tumor diagnosed preoperatively as cT2 was confirmed later to be a pT1 tumor, and three tumors classified initially as superficial type (cT1) were later confirmed to be pT2 or pT3 tumors. The distance from the AV and size were significantly longer (median 11.5 cm *vs* 8 cm; *P* = 0.021) and larger (median 4.6 cm *vs* 3 cm, *P* = 0.039), in patients who underwent incomplete MITAS LE than in those who underwent complete MITAS LE. Spinal anesthesia was used in 235 patients, epidural anesthesia in 1, and general anesthesia in 7. No intraoperative complications occurred, and a stapler was used a mean of 3.6 times (SD 5.7). Table 2 summarizes the operative outcomes.

**Table 1:** Characteristics of the 243 patients who underwent minimally invasive transanal surgery

	Final histology								
		Cancer Grade					NET G1		
Characteristics	All	Tis	T1	T2-	No residual tumor	Adenoma	NET G1	No residual tumor	Others****
(n)	(243)	(82)	(46)	(11)	(3)	(47)	(34)	(18)	(2)
Sex									
Male	140	47	29	5	1	29	16	13	0
Female	103	35	17	6	2	18	18	5	2
Age (y)									
Median (range)	64 (24–92)	69 (45–92)	69 (35–90)	76 (50–85)	59 (46–65)	61 (34–82)	55 (29–72)	56 (26–71)	43 (28–57)
Surgery									
Initial	221	82	46	11	0	46	34	0	2
Additional	22	0	0	0	3	1	0	18	0
Tumor size (mm)									
Median (range)	30 (4–80)**	30 (6–80)	25 (4–60)	40 (15–70)	–	30 (10–70)	8 (3–15)	–	26 (22–30)
≥ 30 mm (%)	97 (40)	41 (50)	18 (39)	9 (82)	0	28 (61)	0	0	1 (50)
≥ 1/3 circle (%)	80 (33)	36 (44)	12 (26)	8 (73)	0	24 (52)	0	0	0
Length from AV* (cm)									
Median (range)	8 (4–20)	8 (4–20)	8 (4–16)	6 (4–15)	5 (5–10)	8 (5–17)	7 (4–12)	7.8 (4–14)	7 (5–9)
Appearance (n)									
Protruded	100	23	19	0	0	22	34	0	2
Superficial	113	59	26	3	0	25	0	0	0
Types 1–5***	9	0	1	8	0	0	0	0	0
Scar	21	–	–	–	3	0	0	18	0

^*^AV: anal verge, **Size excluding tumors with no residual tumor cells histologically

^***^ Types 1–5 according to Japanese Classification of Colorectal, Appendiceal, and Anal Carcinoma

^*^*** One schwannoma and one mucosa-associated lymphoid tissue lymphoma

**Table 2:** Perioperative outcomes of the 243 patients who underwent minimally invasive transanal surgery

Outcomes		Final histology							
		Cancer Grade					NET G1		
	All	Tis	T1	T2-	No residual tumor	Adenoma	NET G1	No residual tumor	Others
	(*n* = 243)	(*n* = 82)	(*n* = 46)	(*n* = 11)	(*n* = 3)	(*n* = 47)	(*n* = 34)	(*n* = 18)	(*n* = 2)
Operative time (min)									
Mean (SD)	27.4 (42.4)	27.6 (10.6)	29.2 (13.4)	41.2 (31.8)	16.3 (3.5)	30.3 (38.2)	17.2 (3.5)	27.3 (4.9)	25 (2.8)
Bleeding volume (mL)									
Mean (SD)	11.0 (17.7)	11.6 (25.5)	9.5 (14.1)	22.6 (73.5)	0 (0)	20.3 (3.5)	1.0 (14.1)	2.8 (7.1)	0 (0)
Complications									
No. of patients (%)	17 (7.0)	6	5	2	2	0	1	1	0
Anastomotic dehiscence	2 (0.8)	0	1	1	0	0	0	0	0
Postoperative bleeding	7 (2.9)	3	2	0	1	0	0	1	0
Spinal headache	6 (2.5)	2	1	1	1	0	1	0	0
Prostatitis	1 (0.4)	0	1	0	0	0	0	0	0
Intramesenteric hematoma	1 (0.4)	1	0	0	0	0	0	0	0
Mortality	0	–	–	–	–	–	–	–	–
En bloc resection									
No. of patients (%)	243 (100)	–	–	–	–	–	–	–	–
Surgical margin negative (R0)									
No. of patients (%)	239 (98.4)	82 (100)	45 (97.8)	9 (81.8)	3 (100)	46 (97.9)	34 (100)	18 (100)	2 (100)

*En bloc* resection of the tumor was completed in all patients. The surgical margin was positive in four patients (1.6%). Of these patients, one with a pT2 tumor and multiple liver metastases, was treated with hepatic arterial infusion chemotherapy; one with a pT3 tumor, diagnosed preoperatively as cT1, underwent APR for the tumor located 4 cm from the AV, and a residual tumor with metastatic lymph nodes (LN) was confirmed; one with a pT1b tumor was followed up for concomitant severe liver cirrhosis; and one with an adenoma was followed up because immediate postoperative endoscopy did not reveal any prominent residual tumor. Of the 243 tumors, 209 were removed by full-thickness excision and 34 by partial wall excision.

Subsequent rectal excision was performed for 19 patients immediately after MITAS LE following confirmation of unfavorable histology (15 of 33 with a high-risk T1 cancer, 3 of 11 with T2 or deeper tumors, and 1 with a T2 NET G1). The remaining 18 patients with high-risk T1 tumors did not undergo further treatment either because they declined or because of comorbidities or advanced age. Among the 11 patients with T2 or deeper tumors, 3 with cT1 tumors found preoperatively underwent subsequent surgery, 2 with accompanying liver metastasis received chemotherapy, and 6 refused any further treatment. Anterior resection was performed for 18 (94.7%) of 19 patients, and 1 patient with a pT3 tumor and a positive margin underwent APR, as described previously. Adhesion around the rectum and mesorectum was mild during subsequent surgery, and the mesorectal fascia of the resected specimen was maintained in all the patients. The final histology of the resected specimen revealed residual tumor of the rectum in one patient with a positive margin after MITAS LE; otherwise, no residual tumor of the rectum was confirmed. Lymph node metastases were confirmed in 3 of the 19 patients. No recurrent disease was identified in these 19 patients who underwent subsequent surgery, during a median follow-up of 108 (range 31–208) months.

Two patients (one withT1 cancer and one with adenoma) were lost to follow-up, and the median follow-up period was 100 (0–301) months. A total 223 patients (91.8%) were followed up for a minimum of 5 years or until death and 115 (47.3%) were followed up for 10 years or until death. Recurrence was identified in 7 (2.9%) of the 243 patients (Table 3), including in 5 (11.9%) of the 42 patients with high-risk T1 tumors and T2 or deeper tumors without liver metastasis. There was no recurrence identified in 95 patients with Tis and low-risk T1 tumors. Local recurrence developed in 3 (6.8%) of 44 patients with high-risk T1 tumors and T2 or deeper tumors (Patients 2, 3 and 4; Table 3). Two of four local recurrences developed at the suture line in patients with a positive margin (Patients 3 and 6; Table 3) within 12 months after MITAS LE. One older patient (Patient 4) with a high-risk T1 tumor was found to have local recurrence outside the suture line. Another patient (Patient 2) was found to have local recurrence as a pelvic mass 53 months after LE and was treated with chemoradiation and extensive surgery without further recurrence. Subsequent surgery was found to be a risk factor for local and/or distant relapse (*p* = 0.002) among the risk factors of age, sex, histopathological characteristics, margin positivity and subsequent surgery, for patients with high-risk T1 tumors and T2 or deeper tumors without liver metastasis.

**Table 3:** Outcomes of seven patients with recurrence after minimally invasive transanal surgery

Patient	Size/Distance From AV (cm)	Final histology	Subsequent surgery	Time to recurrence (mo)	Recurrence site	Treatment	Outcomes (mo), State
1	4.0/6.0	T1b, L1, V0	Refused	10	Lung, Liver, LN*	Chemotherapy	24, Cancer death
2	1.5/8.0	T1b, L1, V0	Refused	53	Local	CRT + APR**	281, Alive, No recurrence
3	2.5/8.0	T1b, L1, V0, Margin (+)	No (liver cirrhosis)	12	Local, LN*	Repeat MITAS × 2	81, Cancer death
4	2.5/8.0	T1b, L1, V1	No (advanced age)	36	Local	Repeat MITAS	85, Senility, No recurrence
5	1.5/10.0	T2, L1, V1	Refused	33	Liver, LN*	Chemotherapy	59, Cancer death
6	5.0/10.0	Adenoma, Margin (+)	No (observation)	8	Local	LAR***	136, Alive, No recurrence
7	1.0/10.0	No NET G1 left	No (observation)	74	Liver, LN*	LAR*** Liver resection Chemotherapy	141, Alive with liver metastasis

^*^LN: Regional lymph node, **APR: abdominoperineal resection, *** LAR: Low anterior resection

**Fig. 2:**
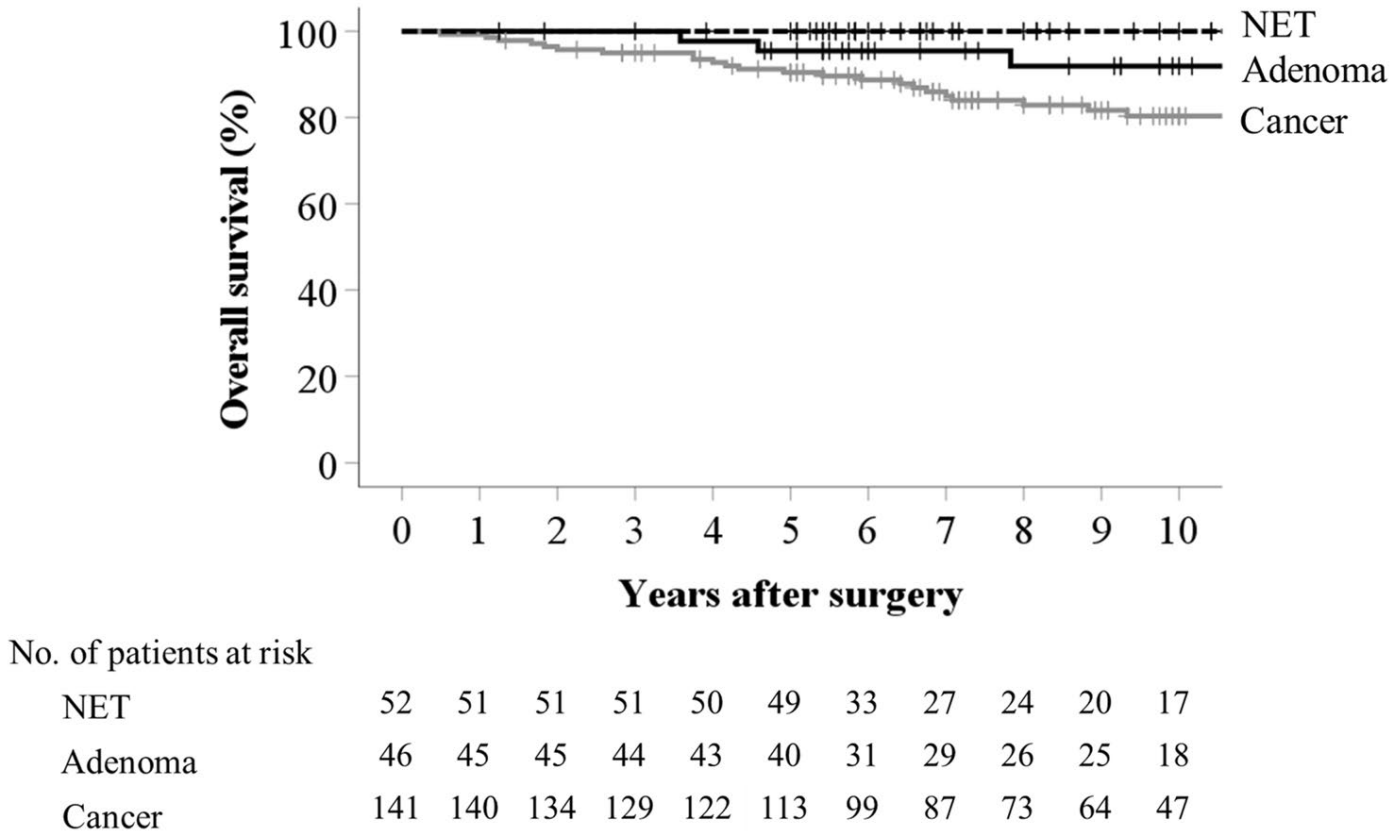
Overall survival of patients with neuroendocrine tumors (NETG1), adenoma, or cancer. One patient with T1 cancer and one patient with adenoma were lost to follow-up

**Fig. 3:**
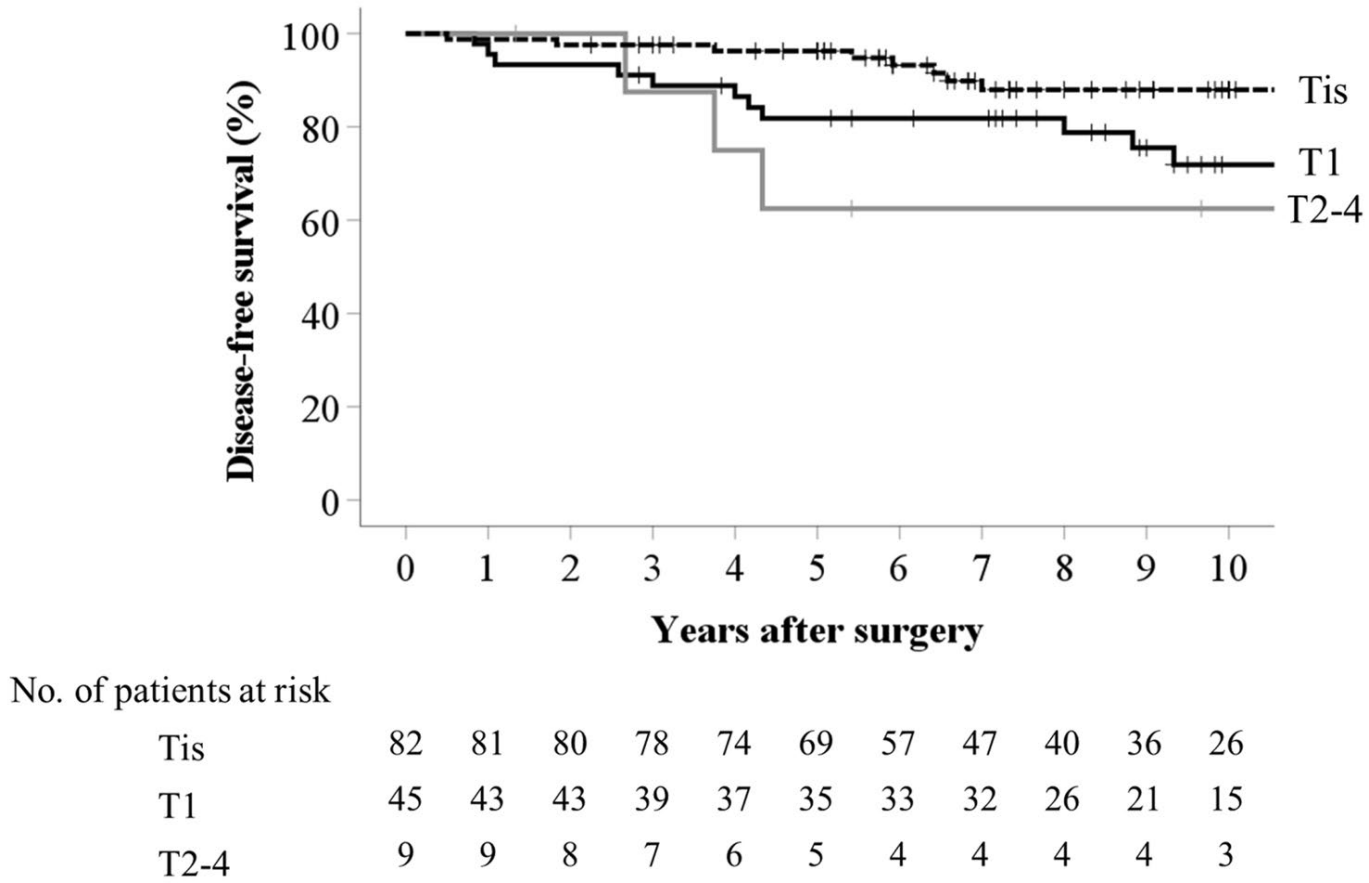
Disease-free survival of patients with Tis, T1, and T2–3 tumors. One patient with T1 cancer was lost to follow-up. Two patients with T2–4 cancer and distant metastases were excluded from the analysis

The 5- and 10-year OS rates were both 100% for patients with NET G1, 95.5% and 91.9% for those with adenoma, and 90.5% and 80.3% for patients with cancer (NET vs cancer, *P* = 0.02), respectively (Fig. 2). The 5- and 10-year OS rates were 84.9% and 68.5% for patients with high-risk T1 tumors and T2 or deeper tumors without liver metastasis, respectively. The 5- and 10-year DFS rates were both 100% for patients with Tis cancer, 90.6% and 90.6% for those with T1 cancer, and 87.5% and 87.5% for those with T2 and deeper cancers, excluding patients with stage IV and no residual cancers, respectively (Tis vs T1 or T2 and deeper, *P* = 0.006 or *P* = 0.002) (Fig. 3). The 5- and 10-year DFS rates were 77.6% and 66.6% for patients with high-risk T1 tumors and T2 or deeper tumor without liver metastasis, respectively. The 5- and 10-year local recurrence-free survival rates were 97.7% and 97.7% for patients with any cancer grade, respectively.

### Outcomes of patients with adenoma and cancer ≥ 3 or < ss3 cm in diameter

Patients with adenomas or cancers (Tis and T1) ≥ 3 cm in diameter (n = 87) or < 3 cm in diameter (n = 87) underwent initial resection by MITAS LE (Table 4). The characteristics of the patients with tumors were equivalent in both groups, except for the size of the tumors. Operative time and bleeding volume were significantly longer and greater, respectively, in the patients with tumors ≥ 3 cm than in those with tumors < 3 cm. However, the surgical margin negative rate, complication rate, recurrence rate, and DFS were equivalent, regardless of tumor size.

**Table 4:** Outcomes of patients with adenoma or cancer with tumors of ≥ 3 cm or < 3 cm

	Tumor ≥ 3 cm (*n* = 87)	Tumor < 3 cm (*n* = 87)	*P* value
Sex, Male/Female (*n*)	49/38	55/32	0.440
Median age, y (range)	68 (34–92)	68 (35–90)	0.942
Median tumor size, mm (SD)	40 (30–80)	20 (4–27)	< 0.001
Median length from AV*, cm (range)	8 (4–20)	8 (4–20)	0.577
Final histology (n)			
Adenoma/Tis /T1	28/41/18	18/41/28	0.114
Mean operative time, min. (SD)	33.5 (12.7)	24.1 (5.7)	0.001
Mean bleeding volume, mL (SD)	19.2 (32.5)	7.8 (19.1)	0.032
Surgical margin negative (R0)			
No. of patients (%)	86 (98.9)	86 (98.9)	0.999
Complications, n (%)			
All	3 (3.4)	8 (9.2)	0.132
Anastomotic dehiscence	0	1	
Postoperative bleeding	1	4	
Follow-up (mo)			
Median (range)	96 (0–262)	107 (0–301)	0.060
Recurrence, n (%)	2 (2.3)	3 (3.4)	
Disease-free survival (%)			0.799
5 years	90.5	94.0	
10 years	83.7	90.5	

^*^AV anal verge

## Discussion

Baatrup et al. [28] reported that tumor size remains a significant predictor for total and cancer-specific survival and that TEMS should not be performed for tumors > 3 cm. However, there is limited evidence for a definitive comparison of outcomes based on different tumor sizes. We found that the characteristics of patients and tumors, operative morbidity, mortality, and oncological outcomes were equivalent among patients, but that MITAS LE was associated with a longer operative time and higher bleeding volume in patients with tumors ≥ 3 cm.

TEMS offers better visualization of and access to more proximal lesions than conventional LE, while TEMS and TAMIS are comparable [3, 8, 16, 29]. A comparison of ESD and TEMS revealed that ESD lesions are more proximal (mean 8.4 cm vs 5.1 cm from the AV) [30]. The size and location of tumors excised by MITAS LE were equivalent to those of tumors excised by ESD, TEMS, and TAMIS [4, 16, 30–32]. Among TAMIS, TEMS, and conventional LE, conventional LE has the shortest operative duration [31], while ESD has a shorter operative time than TEMS (mean: 79.8 min vs 116.6 min) [30]. The mean operative time for ESD is 116 (SD 88) min [32] vs 69.5 (SD 37.9) min for TAMIS [8]. In the present study, the mean operative time was 27.4 (SD 42.4) min for MITAS LE of tumors of equivalent size and height. A circumferential incision was performed in all the pre-existing LE procedures. In MITAS LE, the incision was half as long as when the rectal wall is folded and excised by the stapler, thus saving operative time.

Equivalent postoperative complication rates have been documented for patients undergoing ESD and TEMS (8.0% vs 8.4%) [33] and those undergoing conventional LE and TEMS [29, 34]. Postoperative morbidity was reported in 11% of patients undergoing TAMIS [8]. The postoperative complication rate (7%) associated with MITAS LE was equivalent to that of pre-existing procedures.

*En bloc* resection is performed for 65–76.2% of tumors with conventional LE and 84.6–100% of tumors with TEMS [29, 30, 34, 35]. An equivalent or better *en bloc* resection rate is achieved with TEMS than with ESD [30, 33], whereas TAMIS achieves an *en bloc* resection rate of 95% [8]. A portion of the specimen should be retracted for excision in these procedures, which might induce fragmentation. The specimen was retracted entirely by retraction stitches and excised in MITAS LE, resulting in an *en bloc* resection rate of 100%. R0 resection was performed in 50%–81.1% of patients undergoing conventional LE [34–36] and 80%–90% of those undergoing TEMS [34, 35]. The R0 resection rate varied from 74.6% to 89% in patients undergoing ESD [31, 33] and 93% in those undergoing TAMIS [8]. A superior R0 resection rate (98.4%) was achieved with MITAS LE.

According to various studies, the local recurrence rate ranged from 7 to 21% after LE for T1 lesions and between 2.7% and 6.9% after radical resection, increasing consistently thereafter [1, 3, 5, 18, 37–39]. The 10-year local recurrence rate was 17% in patients undergoing LE for T1 rectal cancers [17] and the 5-year local recurrence rate after TEMS for T1 lesions ranged from 4 to 24% [28, 40, 41]. The rate of local recurrence after TAMIS was 6% and that of distant organ metastasis was 2% for malignant lesions during a mean follow-up of 14.4 months [8]. The local recurrence rate in this series, of 6.5% for T1 lesions and 0% for low-risk T1 and Tis lesions, corresponds to that after radical resection. Furthermore, all local recurrences occurred in patients with a positive margin or high-risk T1 cancer, which means that an appropriate specimen for histological evaluation was obtained by MITAS LE. Local recurrence appeared within 12 months after LE in patients with a positive margin; therefore, intensive follow-up for 1 year is required for patients with a positive margin.

The 5-year DFS rate was worse after conventional LE than after radical surgery for T1 tumors, but equivalent for low-risk T1 tumors [38–40]. The 5-year DFS following TEMS for T1 tumors ranged from 82.4% to 94% [28, 40] and the 3-year DFS after TAMIS for patients with rectal adenocarcinoma was 84% [8]. In our series, the 5- and 10-year DFS for patients with T1 tumors were 90.6% and 90.6%, respectively. Madoff proposed a possible explanation for treatment failure after conventional LE and TEMS, in that both procedures create a raw surface in the mesorectum where tumor cells can be implanted, theoretically [42]. Exfoliated cancer cells were found in the rectum of 33% of patients with T1b and T2 tumors before intersphincteric resection, but in only 1 of 39 sites in the rectum around 13 Tis or T1a tumors [43]. All pre-existing procedures could result in cancer cell implantation from the raw surface created. In contrast, no raw surface is created in MITAS LE following simultaneous excision and anastomosis with a stapler. Swabbing the rectal wall on and around the stapler line was done routinely to prevent implantation into the rectal wall in MITAS LE, which may have accounted for the favorable oncological outcomes in this study.

An equivalent OS rate was reported for patients undergoing LE and radical surgery for T1 and low-risk-T1 tumors, [1, 2, 4, 37, 39], while a better OS was reported after radical surgery than after LE [4, 5, 38, 44]. The 10-year OS rate was 74% for patients undergoing LE for T1 rectal cancers [17]. Thus, MITAS LE accomplished favorable OS for rectal cancer patients.

Worse outcomes have been reported following subsequent rectal excision with TME after LE, in addition to poor specimen quality, increased APR rate, and lower DFS [13, 14, 45, 46]. In the present study, only mild adhesion during subsequent surgery, an intact mesorectal fascia plane, a low APR rate, and no recurrent disease were confirmed following subsequent surgery, possibly because MITAS LE was performed without opening or perforating the rectal wall, and two inverted layers of the rectal wall were excised and anastomosed simultaneously by the stapler.

A disadvantage of MITAS LE is the cost of staplers, as currently, only up to three stapler applications for MITAS LE are covered by Japanese national health insurance. The development of curved staplers could decrease stapler use as a higher number of staplers are needed to excise a round or oval tumor using a straight stapler, to maintain a safe surgical margin. Patients with anal stenosis or a proximally located large tumor are at risk of incomplete MITAS LE. The feasibility of MITAS LE in patients with a high BMI remains to be evaluated.

In this era of skilled endoscopic resection, MITAS LE would be indicated when *en bloc* resection is challenging or for tumors located at the back of the folds. In multimodal treatment, MITAS LE would be a useful method of LE.

A limitation of this study is that it was a retrospective, single-center study. Furthermore, all MITAS LE procedures were performed by experienced colorectal surgeons, thereby restricting the generality of these results.

## Conclusions

For carefully selected patients, MITAS LE is a feasible and safe procedure that allows access to proximal tumors, requires a short operative time, and results in favorable perioperative and long-term oncological outcomes. It is also associated with mild adhesion and an intact TME plane in subsequent surgery, even for tumors ≥ 3 cm.
